# Applying Computerized Adaptive Testing to the Negative Acts Questionnaire-Revised: Rasch Analysis of Workplace Bullying

**DOI:** 10.2196/jmir.2819

**Published:** 2014-02-17

**Authors:** Shu-Ching Ma, Tsair-Wei Chien, Hsiu-Hung Wang, Yu-Chi Li, Mei-Shu Yui

**Affiliations:** ^1^College of NursingKaohsiung Medical UniversityKaohsiungTaiwan; ^2^Nursing DepartmentChi-Mei Medical CenterTainanTaiwan; ^3^Department of Hospital and Health Care AdministrationChia-Nan University of Pharmacy and ScienceTainanTaiwan; ^4^Planning DepartmentChi-Mei Medical CenterTainanTaiwan; ^5^Orthopedics DepartmentChi-Mei Medical CenterTainanTaiwan

**Keywords:** computerized adaptive testing, computer on wheels, classic test theory, item response theory, nonadaptive testing, the Negative Acts Questionnaire-Revised

## Abstract

**Background:**

Workplace bullying is a prevalent problem in contemporary work places that has adverse effects on both the victims of bullying and organizations. With the rapid development of computer technology in recent years, there is an urgent need to prove whether item response theory–based computerized adaptive testing (CAT) can be applied to measure exposure to workplace bullying.

**Objective:**

The purpose of this study was to evaluate the relative efficiency and measurement precision of a CAT-based test for hospital nurses compared to traditional nonadaptive testing (NAT). Under the preliminary conditions of a single domain derived from the scale, a CAT module bullying scale model with polytomously scored items is provided as an example for evaluation purposes.

**Methods:**

A total of 300 nurses were recruited and responded to the 22-item Negative Acts Questionnaire-Revised (NAQ-R). All NAT (or CAT-selected) items were calibrated with the Rasch rating scale model and all respondents were randomly selected for a comparison of the advantages of CAT and NAT in efficiency and precision by paired *t* tests and the area under the receiver operating characteristic curve (AUROC).

**Results:**

The NAQ-R is a unidimensional construct that can be applied to measure exposure to workplace bullying through CAT-based administration. Nursing measures derived from both tests (CAT and NAT) were highly correlated (*r*=.97) and their measurement precisions were not statistically different (*P*=.49) as expected. CAT required fewer items than NAT (an efficiency gain of 32%), suggesting a reduced burden for respondents. There were significant differences in work tenure between the 2 groups (bullied and nonbullied) at a cutoff point of 6 years at 1 worksite. An AUROC of 0.75 (95% CI 0.68-0.79) with logits greater than –4.2 (or >30 in summation) was defined as being highly likely bullied in a workplace.

**Conclusions:**

With CAT-based administration of the NAQ-R for nurses, their burden was substantially reduced without compromising measurement precision.

## Introduction

### Background

Workplace bullying is defined as persistent exposure to interpersonal aggression and mistreatment from colleagues, superiors, or subordinates [1,2]. It is a prevalent problem in the workplace with adverse effects on both victims and organizations [3,4]. Many studies have investigated this problem by determining its frequency, identifying groups at risk in different occupational groups and sectors [5], and addressing prevalence of bullying in different countries and among different occupational groups [6]. However, none of these bullied victim evaluations have applied item response theory (IRT) [7] to assess item functioning of the workplace bullying-related questionnaire [8].

Similarly, no studies have reported results on workplace bullying using IRT-based computerized adaptive testing (CAT) to measure respondents’ bullying exposure, especially in the era of computer technology and when questionnaires have become more integrated in recent years. As of April 24, 2013, 127 articles were found on PubMed by searching the keywords “computer adaptive test” (CAT), 309 with “workplace bullying,” and 106 with “workplace bullying nurse”. It is necessary to investigate whether CAT can be applied to yield the same results as traditional nonadaptive testing (NAT) on a workplace bullying scale for nurses and, thereby, reduce their respondent burden.

### Computer Assessment and Computer Adaptive Testing

From the literature, traditional paper-and-pencil or computer-based surveys (NAT) have a large respondent burden and require respondents to answer all the questions [9]. In contrast, CAT-based testing using IRT can achieve similar measurement precision levels as NAT and is approximately half the length of the test [10-13]. However, most CAT articles, except some [9,14,15], compared CAT to NAT with dichotomous items. Whether polytomously scored items on bullying can also be measured as precisely as dichotomous CAT should be further investigated.

### Rasch Analysis

In classical test theory (CTT), raw scores are usually used as linear interval scale measures for additive latency to assess respondents’ underlying ability. Unfortunately, this is not correct [16,17]; therefore, subsequent statistical analyses can be problematic and incorrect in computing mean, variance, correlation coefficients, or Cronbach alpha [18,19]. In particular, CTT encounters problems when dealing with missing data.

To overcome this obstacle, the IRT-based Rasch model [20] was developed to represent the probabilistic relationship between a person measure and an item difficulty in log-odds units, or logits. A useful scale using the Rasch model should be evaluated by 3 steps (prior tests, Rasch fit statistics, and post hoc tests) suggested by Smith [21] and Tennant and Pallant [22] (details shown in Methods) to verify a single domain. In many articles, authors used Rasch modeling to develop CAT on clinical samples, but none adopted the model testing steps recommended by Smith to verify scales before implementing CAT [9,10,23-26].

### Objectives

First, we used a polytomous Rasch rating scale model to examine the workplace bullying scale for CAT use. Second, we developed an Excel Visual Basic for Applications (VBA) CAT module for comparison with NAT on efficiency, precision, and inference from the data of 300 hospital nurses. Third, similar inferences made by CAT and NAT were conducted in addition to investigating significant differences in work tenure between 2 groups. Fourth, a cutoff point of the studied bullying scale was determined for discriminating persons who were bullied victims with a predicted (individual) probability.

We report the CAT advantages if the precision and inference of results made by CAT and NAT are similar. Several limitations of CAT application will be raised for consideration in future studies.

## Methods

### Study Participants

The study sample was randomly selected and recruited using the last 3 digits of the identification card number from nurses of a 1333-bed medical center in southern Taiwan in the summer of 2010. No incentive for participation was offered. A total of 300 nurses completed 2 effective eligibility scales (shown in the following section) using NAT. This study was approved and monitored by the Research Ethics Review Board of the Chi-Mei Medical Center.

Demographic data collected included gender, work tenure in hospitals of all types, age, marital status, and education level.

### Scales Used

#### Exposure to Bullying

The Negative Acts Questionnaire-Revised (NAQ-R) [27] used in this study has 22 items with 5 response alternatives (1=never, 2=occasionally, 3=monthly, 4=weekly, 5=daily) to measure exposure to workplace bullying within the past 6 months. Victimization from bullying during the past 6 months was additionally measured by a single self-labeling victimization question that was used for determining the cutoff point of the studied bullying scale after bullying measures were obtained. The NAQ-R was professionally translated into Chinese by authors in Taiwan using a back-translation technique (English-Chinese-English). With permission from the author [28], we conducted Rasch analysis to test scale unidimensionality (shown in the dimensionality section), the appropriateness level of the 5-category NAQ-R [29], as well as reporting reliability (Cronbach alpha) and dimension coefficient (DC) [30] using the CTT method.

#### Negative Actions Caused by Bullying

Participants were asked questions about their own personal negative experience of bullying and its impact on 5 areas (physical aspects, psychological aspects, interpersonal relations at work, willingness to work, and quality of work) and they were asked to respond to personal symptoms or emotions (eg, gastrointestinal symptoms, fatigue, loss of appetite, crying, fear, anxiety, no sense of belonging, absenteeism, intent to leave the job, hating work, not being able to concentrate on work, loss of patience when caring for patients, frequent occurrence of abnormalities, low self-esteem, sleep disorders, anxiety, concentration disorders, chronic fatigue, anger, depression, several somatic disorders) [31], all of which were subjective self-judgments (yes=1; no=0) and were evolved into a global scale to verify discriminant validity of the NAQ-R.

### Dimensionality

Tennant and Pallant [22] reported that 3 steps should be applied to assess scale unidimensionality: (1) conduct prior testing using Horn’s parallel analysis [32] to make sure that a single dimension is suitable; (2) use Rasch fit statistics ranging from 0.5 to 1.5 [33,34] to determine the usefulness of the 1-dimensional scaling; and (3) run post hoc tests using the first principal components analysis (PCA) component of Rasch standardized residuals [35] close to zero to inspect the convergent validity, and then performing Smith [36] independent *t* tests to compare estimates of the percentages (<5%, within ±1.96) and verify invariance of Rasch model (details presented in following section).

### Differential Item Functioning

The Rasch rating scale model (used in this study) requires the item estimation to be independent of the subgroups of individuals completing the questions. In other words, item parameters should be invariant across populations [37]. Items not demonstrating invariance are commonly referred to as exhibiting differential item functioning or item bias.

The chi-square test used for detecting the item-trait interaction was computed from a comparison of the observed overall performance of each trait group on the item with its expected performance [38]. Its probability (eg, <.05) reports the statistical probability of observing the chi-square value (or worse) when the data fit the Rasch model. Thus, WINSTEPS table 3.4 was referred to detect differential item functioning items for a significantly different group of person measures [39].

### Computer Adaptive Test Procedures and Features

**Figure 1:**
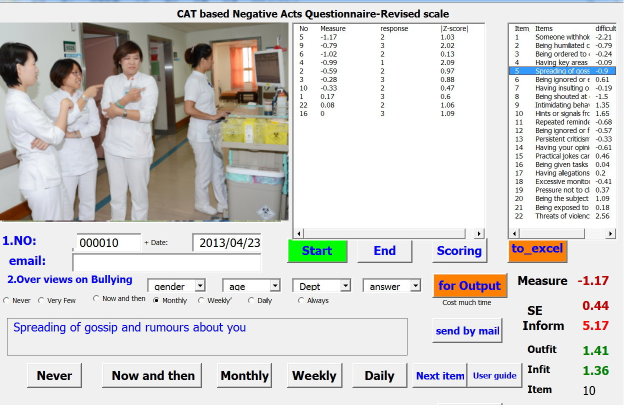
Computer adaptive test applied to Negative Acts Questionnaire-Revised (NAQ-R) in workplace.

**Figure 2:**
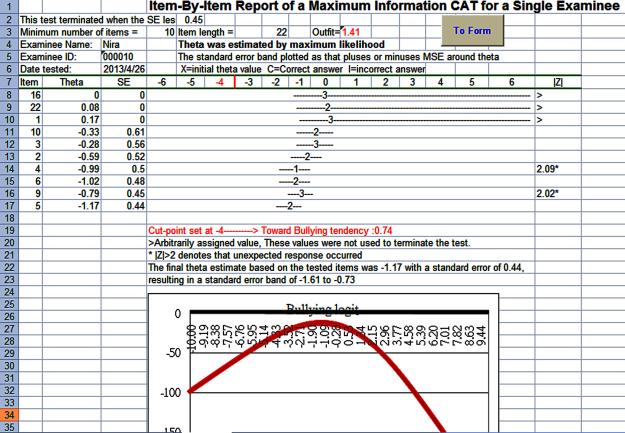
Bullying report produced by Negative Acts Questionnaire-Revised (NAQ-R) computer adaptive test with a maximum likelihood estimation plot.

We ran a VBA module in Microsoft Excel in compliance with rules and regulations of CAT (Figures 1 and 2). Cronbach alpha and Rasch person separation reliability calculated from the NAQ-R of the study were used to determine the CAT termination criterion using the standard error of measurement (SEM=SD × √reliability), whereas Rasch reliability refers to reliability in the previously mentioned SEM formula.

We also set another rule that the minimum number of questions required for completion was 10 (10/22 items on NAQ-R item length=45%) because CAT could achieve similar precision in measurement as NAT with approximately half the length [9-12]. The first question was selected randomly from the 22 items when performing the CAT. The provisional measures were estimated by a maximum log likelihood function using an iterative Newton-Raphson procedure [9,12] after 3 questions were answered without responding similarly and sequentially to either 1 or 5. The next question selected was the one with the most information obtained from the remaining unanswered questions, interacting with the provisional person measures. All responses and their respective consumption time for each nurse were recorded after CAT termination.

### Comparison of Efficiency and Precision

Four indexes between CAT and NAT were compared, including test length (efficiency), estimated measures (precision), time saved (in seconds) per item (efficiency), and the area under the receiver operating characteristic (AUROC) curve (precision).

Accordingly, all person measures based on NAT should be estimated in advance, assuming all 22 items were answered. The following steps were adopted: (1) using the WINSTEPS software [39] to calibrate item and threshold difficulties, and (2) performing the studied dataset of 300 people × 22 items to re-estimate both NAT (through all 22 items) and CAT (by less than 22 items) measures using the CAT Excel-VBA module.

### Comparison of Groups by Making Inferences

We compared the prediction effects of CAT and NAT on the 4 indexes by regressing person measures, respectively, on (1) the global symptom score, and (2) differences in demographic characteristics (eg, age, work tenure, and marital status) and in self-judgments (victimization from bullying during the past 6 months).

### Statistical Analysis

For all statistical analyses, SPSS software for Windows version 12 (SPSS, Chicago, IL, USA) was used. The CAT and NAT person measures were compared using the Pearson correlation coefficient. Test length (efficiency) and estimated measures (precision) were compared by paired *t* tests. Time saved per item (efficiency) in favor of CAT was computed by a margin of 25.07 seconds (SD 16.04; range 12-43). The total time saving from NAT to CAT was computed by the formula: time saved per item (25.07) multiplies both item lengths shortened by CAT (eg, 2109 items in total), and the sample size (N=300).

The AUROC (precision) was calculated by both Rasch-transformed logit scores and the single self-labeled victimization question from bullying (bullied=1; not bullied=0) to determine a cutoff point with 95% confidence intervals (CIs), sensitivity, and specificity. The criterion of alpha=.05 was considered statistically significant. Horn’s parallel analysis was performed using an online calculator [40] that is based on the literature [32,41].

## Results

### Overview

Two age groups (separated by a cutoff point of 30 years) were contrasted on demographic characteristics (eg, self-labeled victimization from bullying, gender, work tenure within the study hospital, work tenure in health care, marital status, and education level). As seen in Table 1, the prevalence of bullying within the study hospital was 24.0% (72/300). CAT and NAT measures were highly correlated (*r*=.97)

**Table 1:** Demographic characteristics of participants (N=300).

Characteristics		Age (years), n (%)		Total, n
		<30	≥30	
Age		109 (36.3)	191 (63.7)	300
**Bullied or not**				
	No	84 (36.8)	144 (63.2)	228
	Yes	25 (34.7)	47 (65.3)	72
**Gender**				
	Male	1 (25.0)	3 (75.0)	4
	Female	108 (36.5)	188 (63.5)	296
**Work tenure in the hospital (years)**				
	≤3	50 (92.6)	4 (7.4)	54
	3-6	47 (69.1)	21 (30.9)	68
	6-9	11 (16.2)	57 (83.8)	68
	>9	1 (0.9)	109 (99.1)	110
**Work tenure in health care (years)**				
	≤3	46 (95.8)	2 (4.2)	48
	3-6	47 (83.9)	9 (16.1)	56
	6-9	15 (21.7)	54 (78.3)	69
	>9	1 (0.8)	126 (99.2)	127
**Marital status**				
	Married	22 (16.7)	110 (83.3)	132
	Unmarried	87 (51.8)	81 (48.2)	168
**Education diploma**				
	Undergraduate	109 (37.7)	180 (62.3)	189
	Postgraduate	—	11 (100)	11

### Dimensionality

**Figure 3:**
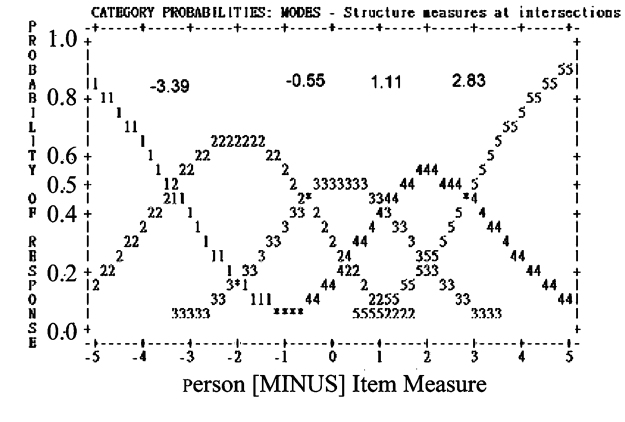
Threshold step difficulties monotonically increasing for the Negative Acts Questionnaire-Revised (NAQ-R).

**Figure 4:**
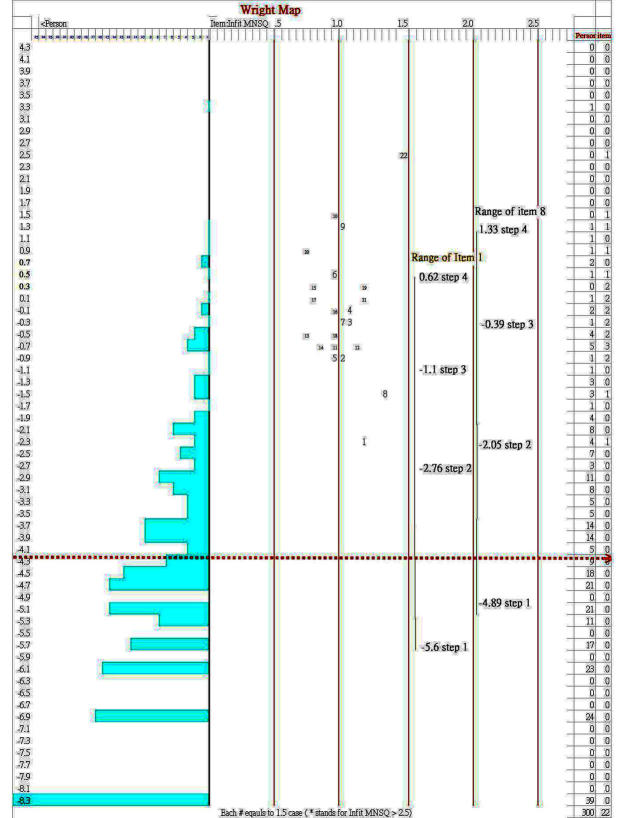
Variable map for person and item dispersion on Rasch logit scores.

The NAQ-R can be considered unidimensional given that (1) one factor was extracted by parallel analysis; (2) all infit and outfit mean squares for the 22 items were in a range of 0.5 to 1.5 (shown in Table 2); (3) item loadings from the Rasch PCA of residuals on the first contrast were closely clustered within 0.6 or near 0.6; PTME (ie, point measure regarding the Pearson correlation between the observations of an item and the item difficulties that is similar to factor loading) between 0.48 and 0.78; Rasch person separation reliability=0.90, Cronbach alpha=.98 (>.70), DC=0.89 (>0.70), and Smith’s *t* test of proportions [36] reach zero outside the range ±1.96 (ie, all persons’ paired *t* values were within ±1.96). In addition, category structure for the NAQ-R should display the monotonically increasing threshold (-3.39, -0.55, 1.11, and 2.83 logits; Figure 3) with the Linacre’s guidelines [29] to improve the utility of the resulting measures. The absence of differential item functioning suggests good support for measurement invariance. The range of threshold difficulties for those least difficult items 1 and 8 are shown as examples in 2 columns on the right-hand side in Figure 4, indicating that item difficulties cannot sufficiently cover all the person measures with mild or nonbullied symptoms shown on the left bottom in Figure 4 using the NAQ-R scale.

**Table 2:** One factor extracted from the Negative Acts Questionnaire-Revised (NAQ-R) scale with mean square between 0.50 and 1.50.

During the last 6 months, how often have you been subjected to the following negative acts in the work place?		Item		Mean square		Rasch
		Delta	PTME	Infit	Outfit	Loading
**Work-related bullying**						
	19. Pressure not to claim something to which by right you are entitled	0.37	0.68	1.22	0.88	–0.26
	21. Being exposed to an unmanageable workload	0.18	0.67	1.22	1.12	–0.27
	16. Being given tasks with unreasonable deadlines	0.04	0.71	0.94	0.95	–0.21
	3. Being ordered to do work below your level of competence	–0.24	0.68	1.11	1.10	0.59
	18. Excessive monitoring of your work	–0.41	0.77	0.93	0.84	–0.43
	14. Having your opinions ignored	–0.61	0.75	0.87	0.92	–0.45
	1. Someone withholding information which affects your performance	–2.21	0.73	1.21	1.28	0.47
**Person-related bullying**						
	10. Hints or signals from others that you should quit your job	1.65	0.60	0.96	0.76	0.03
	20. Being the subject of excessive teasing and sarcasm	1.09	0.69	0.75	0.63	–0.30
	6. Being ignored or excluded	0.61	0.68	0.93	0.90	0.17
	15. Practical jokes carried out by people you don’t get along with	0.46	0.72	0.81	0.65	–0.32
	17. Having allegations made against you	0.20	0.73	0.81	0.67	–0.32
	4. Having key areas of responsibility removed or replaced with more trivial or unpleasant tasks	–0.09	0.69	1.12	1.08	0.31
	7. Having insulting or offensive remarks made about your person, attitudes or your private life	–0.19	0.72	0.99	0.90	0.14
	13. Persistent criticism of your errors or mistakes	–0.33	0.78	0.77	0.65	–0.49
	12. Being ignored or facing a hostile reaction when you approach	–0.57	0.72	1.17	1.04	–0.34
	11. Repeated reminders of your errors or mistakes	–0.68	0.76	0.95	0.90	–0.26
	2. Being humiliated or ridiculed in connection with your work	–0.79	0.72	1.07	0.98	0.59
	5. Spreading of gossip and rumors about you	–0.90	0.75	0.97	0.96	0.31
**Physically intimidating bullying**						
	22. Threats of violence or physical abuse or actual abuse	2.56	0.48	1.49	0.56	–0.12
	9. Intimidating behaviors such as finger-pointing, invasion of personal space, shoving, blocking your way	1.35	0.61	1.06	0.97	0.11
	8. Being shouted at or being the target of spontaneous anger	–1.50	0.71	1.37	1.36	0.25
Minimum		–2.21	0.48	0.75	0.56	–0.49
Maximum		2.56	0.78	1.49	1.36	0.59

### Comparison of Efficiency and Precision

The CAT required substantially fewer items than NAT (*P*<.001). NAT required all 300 participants to respond to all 22 items, yielding 6600 responses. In CAT, only 4491 responses were required, meaning that each nurse answered 14.97 questions on average. As compared to NAT, CAT provided an efficient gain in test length of 0.32, calculated as 1–(ratio of total responses by CAT and NAT), or 1–(4491/6600).

For precision of measurement, person measures from CAT did not statistically differ from those from NAT (*P*=.14). The total time saving from NAT to CAT was 52,848 seconds (25.07×7.03×300) or 14.68 hours.

Cutoff point values were >–4.2 logits (approximately >30 in traditional summation); AUROC (95% CI), sensitivity, and specificity were found to be similar between CAT and NAT.

### Similar Results in Making Inferences

Rasch logit measures (*x*) of CAT and NAT can yield similar response slope parameters to predict the global scores (*y*) for negative actions. A 2-way ANOVA revealed that person measures only differed in groups of the bullied and the nonbullied as well as groups with job tenure of less than and more than 30 years (in the study hospital) (Table 3).

**Table 3:** Similar inferences made by computer adaptive testing (CAT) and nonadaptive testing (NAT).

Demographic characteristics			NAT			CAT		
			Mean square	*F*_1,1_	*P*	Mean square	*F*_1,1_	*P*
**Age**								
	Age		0.76	0.13	.72	1.21	0.21	.65
	Victim		265.60	46.73	<.001	326.19	55.14	<.001
	Age × victim		10.53	1.85	.18	21.87	3.69	.06
	Residual		5.68			5.92		
	**Bullied in logit**							
		Yes	–3.09	—		–2.86	—	
		No	—	–5.41		—	–5.42	
**Work tenure 1 (in this hospital)**								
	Tenure		23.59	4.18	.11	30.96	5.26	.02
	Victim		267.53	47.41	<.001	327.25	55.56	<.001
	Tenure × victim		6.53	1.16	.28	14.99	2.55	.11
	Residual		5.64			5.89		
	**Tenure (years) in logit**							
		<6	–3.68	—		–3.68	—	
		≥6	—	–4.46		—	–4.46	
**Work tenure 2 (in health care)**								
	Tenure		16.22	2.86	.09	22.52	3.81	.05
	Victim		243.25	42.94	<.001	307.68	51.96	<.001
	Tenure × victim		2.75	0.49	.49	9.95	1.68	.19
	Residual		5.67			5.92		
**Marital status**								
	Marry		0.29	0.05	.82	0.05	0.01	.93
	Victim		259.03	45.31	<.001	306.54	51.15	<.001
	Marry × victim		0.57	0.10	.75	0.44	0.07	.79
	Residual		5.72			5.99		

## Discussion

### Key Findings

The results from this study indicate that the 22-item NAQ-R is considered unidimensional. The CAT is up to 32% more efficient for answering questions and achieved similar precision and inferences in measurements as did NAT. A cut point of >–4.2 logits (or >30 in summation) with AUROC 0.75 (95% CI 0.68-0.79) was determined for future use in workplace bullying surveys. The prevalence of bullying for the study sample was 0.24.

### What This Adds to What Was Known

Consistent with the literature [9-12], the efficiency of CAT over NAT was supported. We confirm the CAT-based NAQ-R requires significantly fewer questions to measure victimization from workplace bullying than NAT without compromising its measurement precision.

### What It Implies and What Should Be Changed?

#### Easy to Detect Unexpected Responses

The CAT module can help us efficiently and precisely gather responses from nurses and it was technically applicable. Outfit mean square values of 2.0 or greater can be used to examine whether responses are distorted or abnormal. That is, much more unexpected responses deemed to occur because of possibly careless, mistaken, or awkward endorsement were found in the measurement [9,10,29] (eg, nurse A gained outfit 1.41 and gave unexpected responses on items 4 and 9 as shown in Figure 2). It is easier to detect problematic responses by using CAT than CTT. Multimedia Appendix 1 is a CAT module that can be downloaded and practiced by interested readers.

#### Steps to Detect Scale Dimensionality Used for Computer Adaptive Testing

Some studies [2,3,5,8] reported that there were 2 or 3 factors extracted from the NAQ-R because it used the eigenvalue greater than 1 (K_1_) rule to extract a number of factors. A number of studies have shown that the K_1_ rule is inaccurate and tends to overfactor [32,42,43]. In contrast, Zwick and Velicer’s [44] comparison concluded that parallel analysis was the most accurate evaluation method and it was correct 92% of the time (greater than 22% using K_1_). That explained why the factor determined using the parallel analysis method in the present study was different from others.

We also uniquely examined it using Smith’s [21] recommended other 2 steps (Rasch analyses shown in Methods) to detect scale dimensionality. Compared to the traditional way of using either parallel analysis or Kayser’s rule to detect the number of factors, Rasch-based analysis is superior in studying the dimensionality of a given instrument (eg, infit and outfit criteria and PCA residuals). Accordingly, the CAT module can be implemented after the scale unidimensionality and item difficulties have been determined using the Smith’s 3 steps and Rasch analysis.

#### Cutoff Point Recommended for Determining Bully Victims

The AUROCs (0.74 and 0.75 for NAT and CAT, respectively) were not as high as expected (>0.80). It might be acceptable in social science when it is greater than 0.70 because the single self-labeled victimization question (bullied=1; not bullied=0) might be subjectively answered with some bias by examinees’ personal perception of bullying.

Regarding another issue that the cutoff point of –4.2 logits is too low to be confident in the lower 24% prevalence of bullying for the study sample, we can see the visualized person and item map in Figure 4. The person sample is not dispersed as normal (with mean 0 and SD 1) as we expected, so that most nurses earn low Rasch-transformed scores. It is because items on the top right-hand side are presented as difficult for nurses to respond to.

In addition, we can use individual Rasch-transformed logit scores to predict their probability of the bullied victimization using the formula: probability=exp(theta–delta)/(1+exp(theta–delta)), where theta=person measure and delta=item difficulty at cutoff point. For instance, a person with –1.5 logits in bully measurement will present his/her probability at 0.94, calculated as exp(–1.5–(–4.2))/(1+exp(–1.5–(–4.2))), where the item difficulty at cutoff point=–4.2 and the specified person measure=–1.5). The 95% confidence intervals are determined by the dispersion of the person’s measured error (ie, the value of 1.96×SE transformed to the previously mentioned probability formula).

### Strengths of This Study

#### Using Computer Adaptive Testing to Endorse the NAQ-R

There are 2 major types of standardized assessments in clinical settings [45]: (1) a lengthy questionnaire that requires significant amounts of time and training for administration to achieve a precise assessment, and (2) a rapid, short-form one that briefly screens for the most common symptoms using cutoff points to determine degrees of impairment [46,47]. CAT has the advantages of both types: precision and efficiency. This paper used the Rasch rating scale model (instead of dichotomy or Rasch partial credit model) to design CAT and then applied it to endorse the NAQ-R. We conducted an actual CAT survey procedure (see the module in Multimedia Appendix 1) instead of CAT simulation as other published studies.

#### Detecting the Appropriateness of Level of Scaling

If the item threshold difficulties (calibrated by Rasch rating scale model) collapsed, categories should be combined to be more efficient for respondents to answer [29]. Unlike NAQ-R on which the responses “about weekly” and “about daily” were subjectively thrown together into one category [48], this study used the Rasch model for detecting the appropriateness of level of scaling [49].

#### Unique Features

Although the efficiency of a CAT has been well validated in the literature, the findings of this study do not appear to contribute any important information on the CAT approach. In this study, 2 unique features were reported to readers: (1) the 22-item polytomous NAQ-R is suited for CAT administration in practice, and (2) the module of animation CAT presented in Multimedia Appendix 1 is available for interested readers to practice, which is rare in any previously published articles.

### Limitations of the Study

#### Issues for Further Consideration in Future Research

Several issues should be considered more thoroughly in further studies. First, few male nurses were included in the sample so that the differential item functioning for gender could not be identified by Rasch analysis. Second, there is potential sampling bias in this study. More studies are needed to assess the generalizability of the study with different samples and in different institutes using the Chinese version of NAQ-R. Third, the prevalence of bullying in this study hospital was 24%, higher than seen in studies of Japanese nurses (19%) [8], Italian employees (15.2%) [50], and general service workers in general services (from 2% to 17%) [51]. Fourth, more objective estimates of the prevalence for bullying based on the Leymann criterion [52] is worthy of carrying out in future because the self-labeling approach in this study might produce some biases [50].

#### Computer Adaptive Testing Stopping Rules Used in This Study

The CAT stopping rules are usually determined by SEM and/or no more than a specific number of items needed for achieving both precision and fast assessment. We applied the former and set minimal items for an acceptable level of person conditional reliability in CAT results.

In Figure 2, we demonstrated a CAT example terminated at SE <0.44, calculated as √(1–0.80), where reliability is set at 0.80, instead of 0.32, calculated as √(1–0.90), where reliability is set at 0.90. If using the latter criterion of 0.32, the item length in CAT will approach the total of 22 items. One way to improve the CAT efficiency and precision is to add more easy bullying questions to the scale (see Figure 4), especially for item difficulties located around the cutoff of –4.2 logits to increase the power of diagnostic discrimination for the bullied victims. Another way is to temporarily lower the acceptable level of precision to 0.80 reliability as was done in this study.

#### Lenient Criterion to Support a Good Model-Data-Fit

According to the literature [34], the range of 0.6 and 1.4 is recommended for rating scales (Likert/survey). Item 22 has a slightly high value of infit mean square error (mean square error=1.49) which is <1.5, but a lower outfit mean square error of 0.56. The high value of infit mean square error suggests that those nurses with highly negative actions caused by bullying have a sensitive misfit to item 22, but will not be influenced by the too-low cutoff score at -4.2 logits. In contrast, the low outfit mean square error shown in Table 2 indicates that item 22 has a good model-data-fit for most general nurses. WINSTEPS’ guidelines [33] supports that a mean square error >1.5 suggests a deviation from unidimensionality in the data. The other 2 model testing steps Smith recommended also verified that the NAQ-R 22-item scale is unidimensional and suggests that it suits CAT administration.

### Applications

#### Developing an Online Computer Adaptive Testing System

Many issues should be further explored in the future, including studies addressing the limitations noted previously and subsequently. For example, the CAT module should be extended to the Internet for easy use so that the NAQ-R can be administered in more workplaces.

#### Applying the Animation Computer Adaptive Testing Module

One of the important advantages of CAT scoring is that the item pool for the 22-item NAQ-R can be expanded to match a wide range of participants covering different kinds of bullied workers without changing the module and measurement accuracy. The CAT users may also expand the NAQ-R item pools or replace them with other kinds of workplace bullying scales. It must be noted that (1) overall (ie, on average) and step (threshold) difficulties of the questionnaire must be calibrated in advance using Rasch analysis, (2) pictures and audio files for each question shown in the CAT Excel-module should be well-prepared and put in an appropriate folder that can be shown simultaneously to correspond to questions for the animation CAT module, and (3) pictures and audio files included in the CAT need to match original meaning of the items as much as possible to avoid distorting the validity of the scale.

If readers would like to conduct Rasch partial credit model for the NAQ-R, the distinct threshold step difficulties across items should be reset in specific Excel cells in Multimedia Appendix 1.

#### More Items Added to Decrease Standard Error

We described the category structure in Figure 3 displaying the NAQ-R monotonically increasing threshold (–3.39, –0.55, 1.11, and 2.83 logits). The Rasch rating scale model indicates each item has a common threshold difficulty. Therefore, the overall difficulties (ie, delta in Table 2) for each item plus the threshold step difficulties (eg, items 1 and 8 in Figure 4) form its own range of item difficulties, and only items 5, 8, and 1 with difficulty ranges include the cutoff point at –4.2 logits. To decrease the person’s measured error (ie, SE), we suggest the NAQ-R 22-item scale should add more easy items in the future to increase individual person conditional reliability.

### Conclusion

The CAT-based NAQ-R forming a unidimensional construct reduces respondents’ burden without compromising measurement precision and increases endorsement efficiency. The computer module developed by the authors is recommended for assessing workers with scores beyond a cut point of >–4.2 logits (or >30 in summed score), who should be treated with more concern as soon as possible at an earlier stage.

## Multimedia Appendix 1

Excel VBA CAT module offered to interested readers for own practice (bullying.zip).

## Abbreviations

AUROC: area under the ROC
CAT: computerized adaptive testing
CTT: classical test theory
DC: dimension coefficient
IRT: item response theory
MNSQ: mean square residual
SEM: standard error of the mean
NAQ-R: Negative Acts Questionnaire-Revised
NAT: nonadaptive testing
ROC: receiver operating characteristic
VBA: Visual Basic for Applications
