# Supplementary figures and images for: Applying Computerized Adaptive Testing to the Negative Acts Questionnaire-Revised: Rasch Analysis of Workplace Bullying
Source: J Med Internet Res. 2014 Feb 17;16(2):e50. doi: 10.2196/jmir.2819 (PMC3958675; doi:10.2196/jmir.2819)

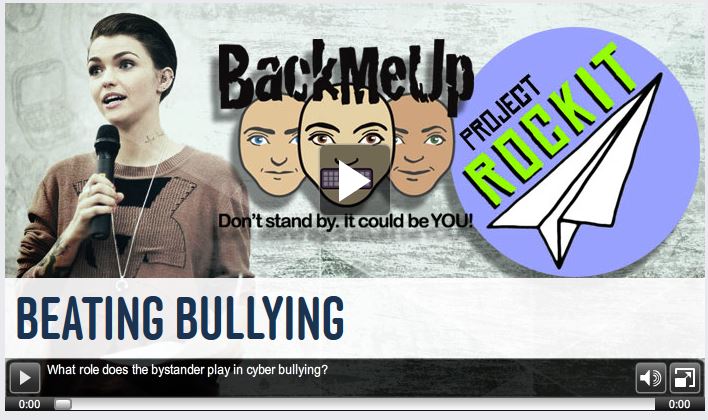

Supplement: Supplementary file 1 [file jmir_v16i2e50_app1.zip › bullying/1.jpg]

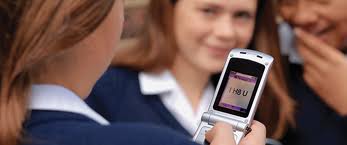

Supplement: Supplementary file 1 [file jmir_v16i2e50_app1.zip › bullying/10.jpg]

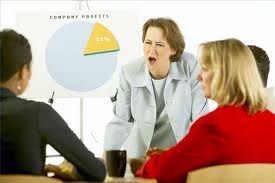

Supplement: Supplementary file 1 [file jmir_v16i2e50_app1.zip › bullying/11.jpg]

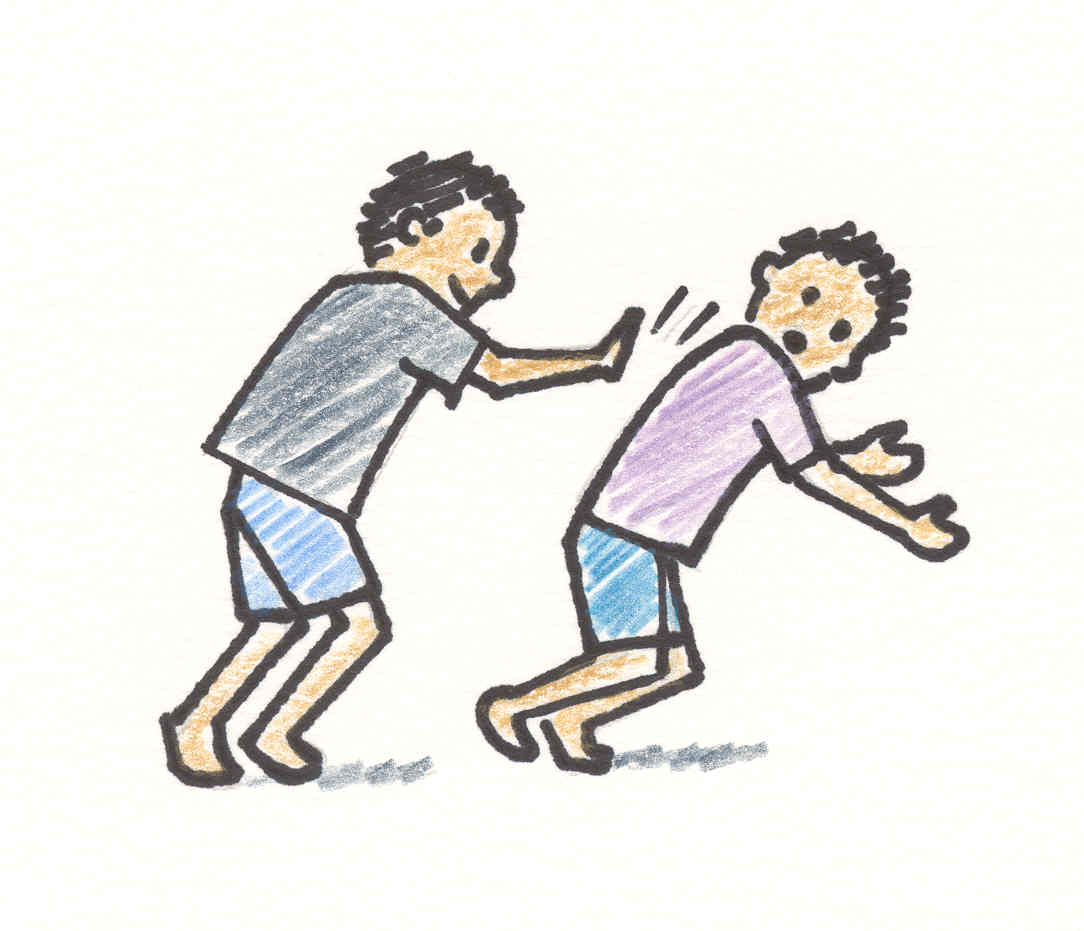

Supplement: Supplementary file 1 [file jmir_v16i2e50_app1.zip › bullying/12.jpg]

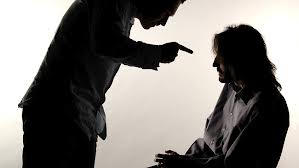

Supplement: Supplementary file 1 [file jmir_v16i2e50_app1.zip › bullying/13.jpg]

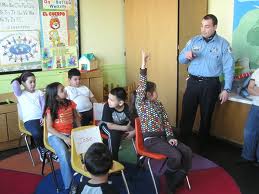

Supplement: Supplementary file 1 [file jmir_v16i2e50_app1.zip › bullying/14.jpg]

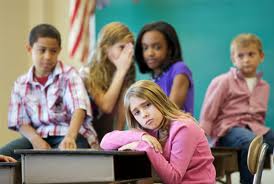

Supplement: Supplementary file 1 [file jmir_v16i2e50_app1.zip › bullying/15.jpg]

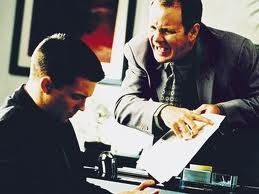

Supplement: Supplementary file 1 [file jmir_v16i2e50_app1.zip › bullying/16.jpg]

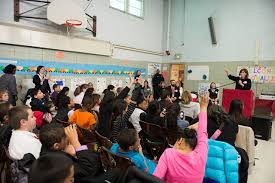

Supplement: Supplementary file 1 [file jmir_v16i2e50_app1.zip › bullying/17.jpg]

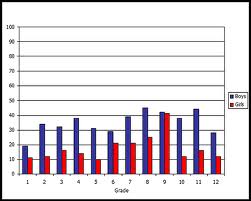

Supplement: Supplementary file 1 [file jmir_v16i2e50_app1.zip › bullying/18.jpg]

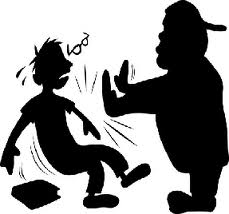

Supplement: Supplementary file 1 [file jmir_v16i2e50_app1.zip › bullying/19.jpg]

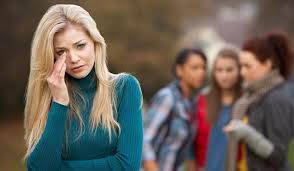

Supplement: Supplementary file 1 [file jmir_v16i2e50_app1.zip › bullying/2.jpg]

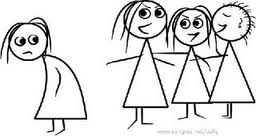

Supplement: Supplementary file 1 [file jmir_v16i2e50_app1.zip › bullying/20.jpg]

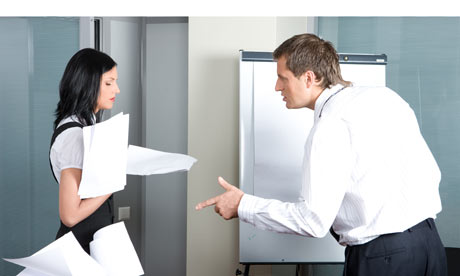

Supplement: Supplementary file 1 [file jmir_v16i2e50_app1.zip › bullying/21.jpg]

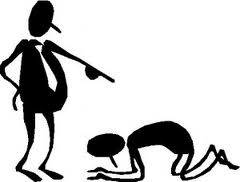

Supplement: Supplementary file 1 [file jmir_v16i2e50_app1.zip › bullying/22.jpg]

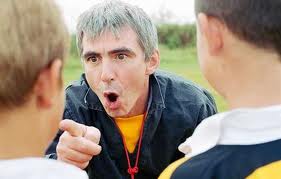

Supplement: Supplementary file 1 [file jmir_v16i2e50_app1.zip › bullying/3.jpg]

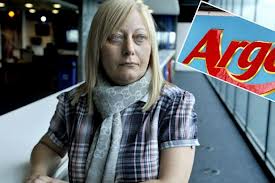

Supplement: Supplementary file 1 [file jmir_v16i2e50_app1.zip › bullying/4.jpg]

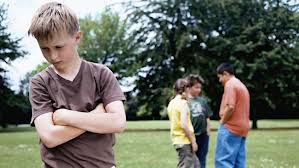

Supplement: Supplementary file 1 [file jmir_v16i2e50_app1.zip › bullying/5.jpg]

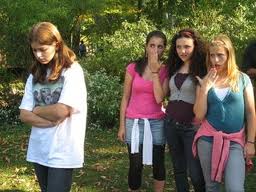

Supplement: Supplementary file 1 [file jmir_v16i2e50_app1.zip › bullying/6.jpg]

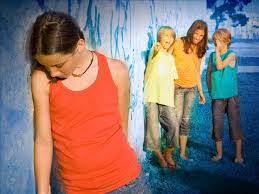

Supplement: Supplementary file 1 [file jmir_v16i2e50_app1.zip › bullying/7.jpg]

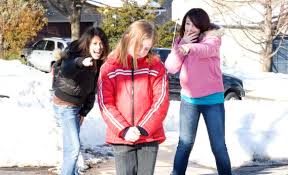

Supplement: Supplementary file 1 [file jmir_v16i2e50_app1.zip › bullying/8.jpg]

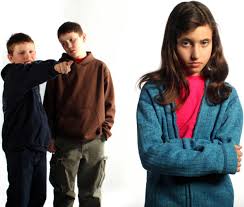

Supplement: Supplementary file 1 [file jmir_v16i2e50_app1.zip › bullying/9.jpg]

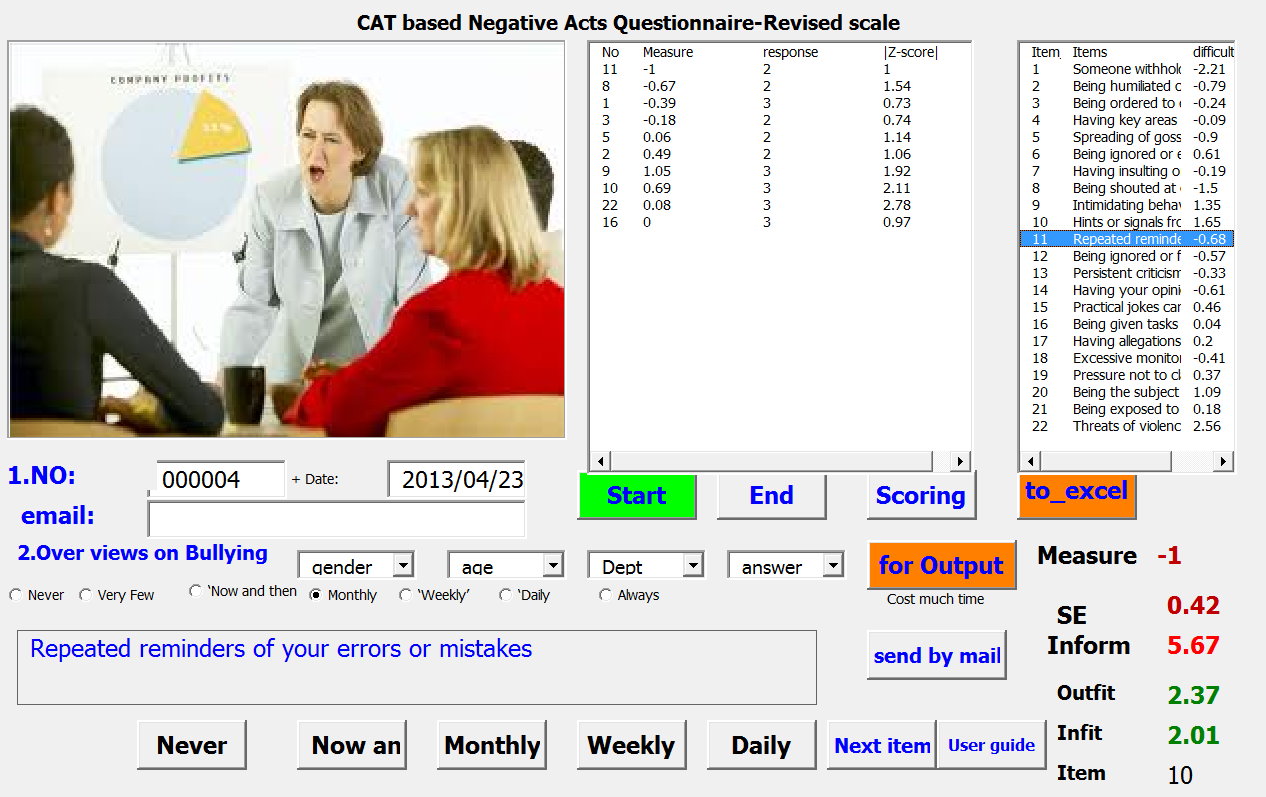

Supplement: Supplementary file 1 [file jmir_v16i2e50_app1.zip › bullying/cat.jpg]

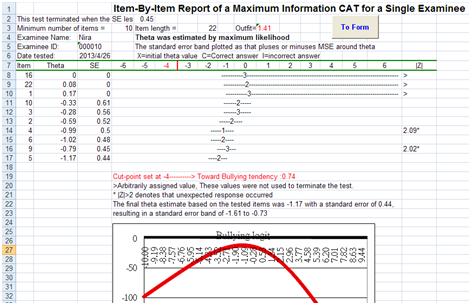

Supplement: Supplementary file 1 [file jmir_v16i2e50_app1.zip › bullying/Figure2.JPG]

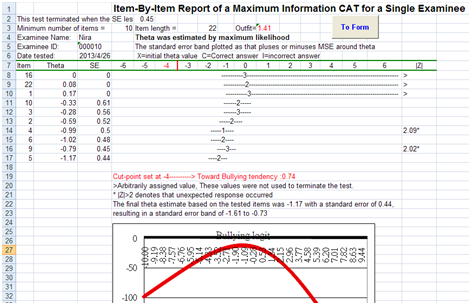

Supplement: Supplementary file 1 [file jmir_v16i2e50_app1.zip › bullying/Figure2.TIF]

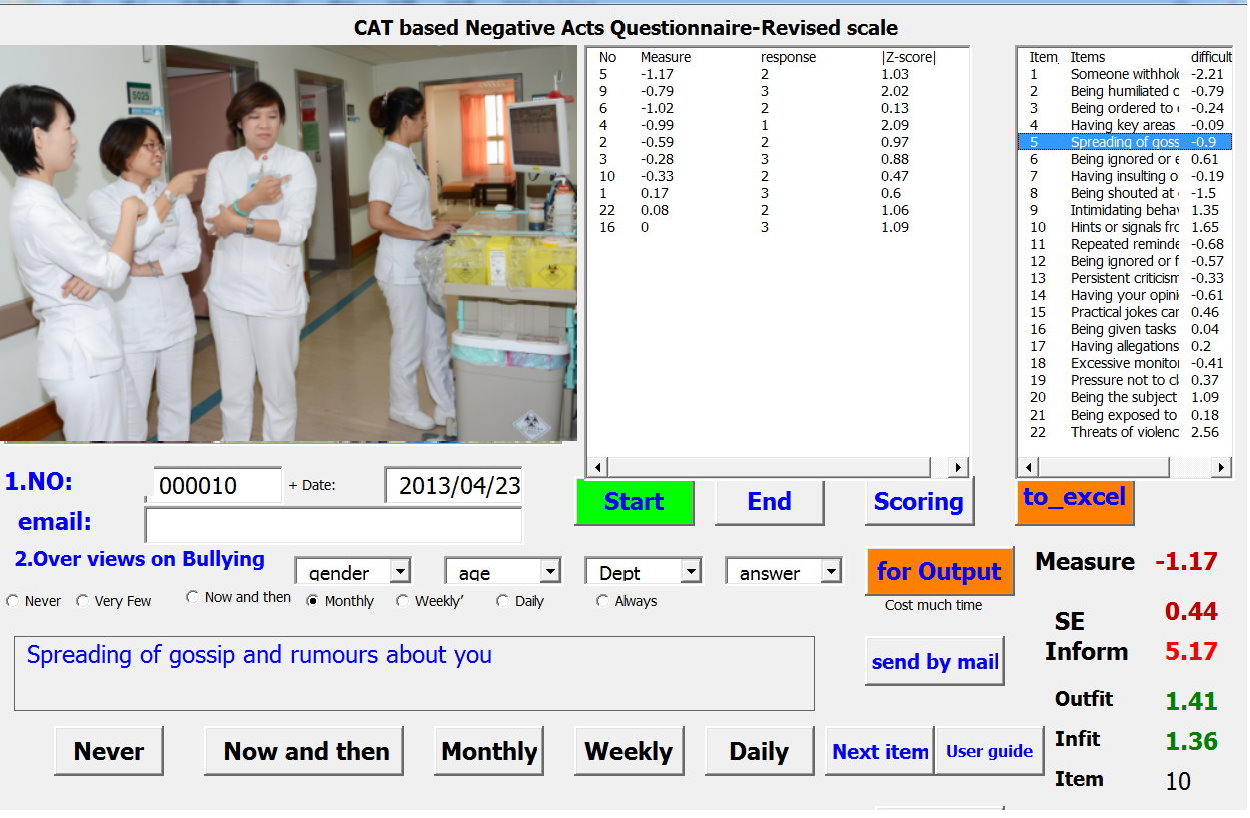

Supplement: Supplementary file 1 [file jmir_v16i2e50_app1.zip › bullying/gossip.jpg]

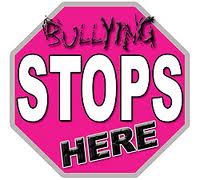

Supplement: Supplementary file 1 [file jmir_v16i2e50_app1.zip › bullying/nopic.jpg]

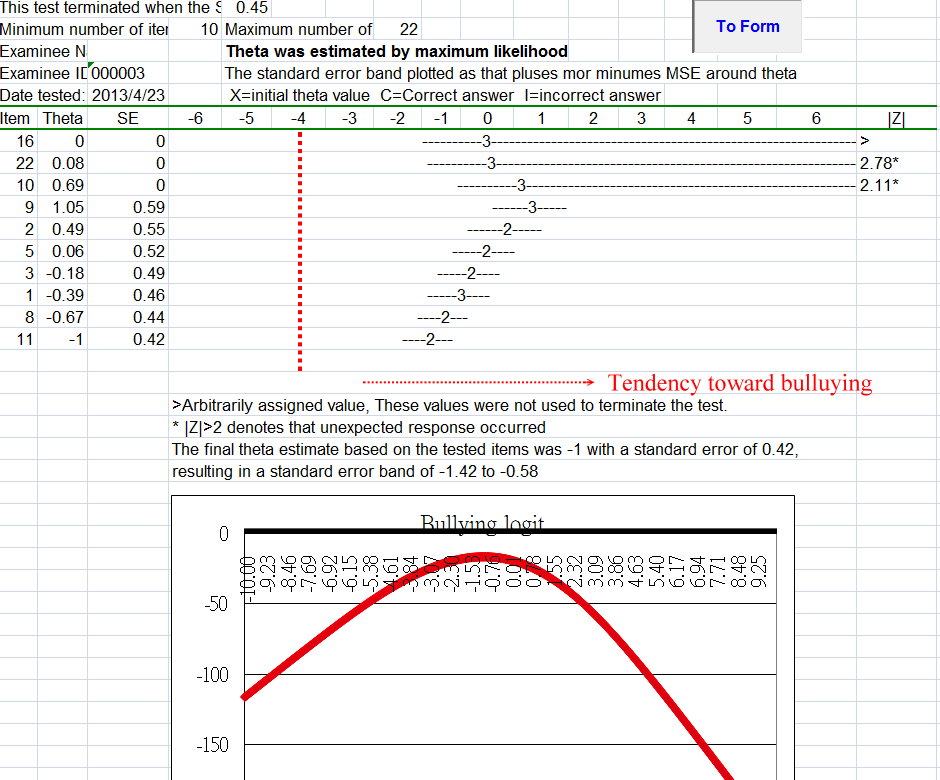

Supplement: Supplementary file 1 [file jmir_v16i2e50_app1.zip › bullying/report.jpg]

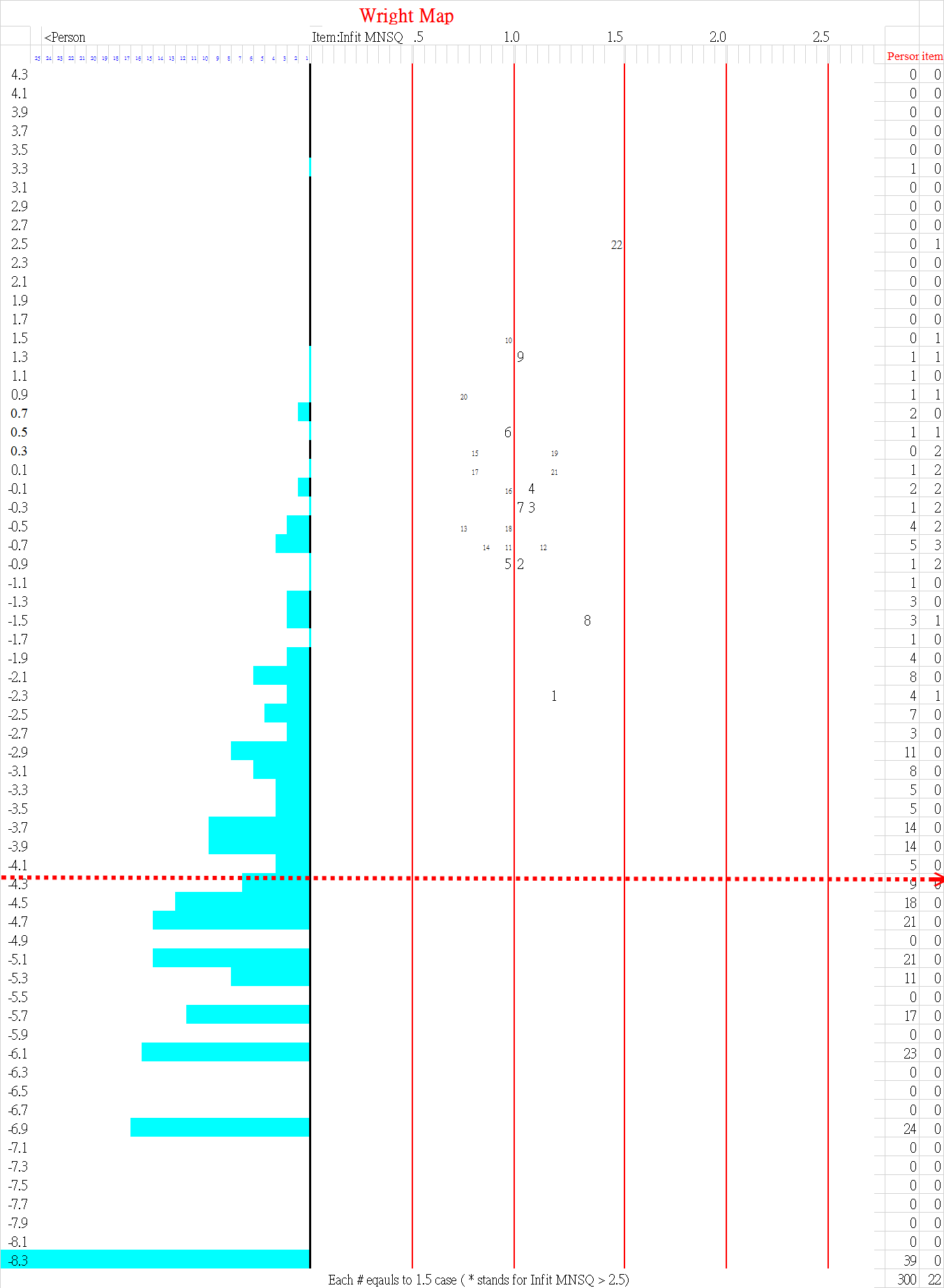

Supplement: Supplementary file 1 [file jmir_v16i2e50_app1.zip › bullying/wrightmap.tif]
